# Supplementary figures and images for: Insulator-based dielectrophoresis-assisted separation of insulin secretory vesicles
Source: eLife. 2024 Aug 27;13:e74989. doi: 10.7554/eLife.74989 (PMC11349295; doi:10.7554/eLife.74989)

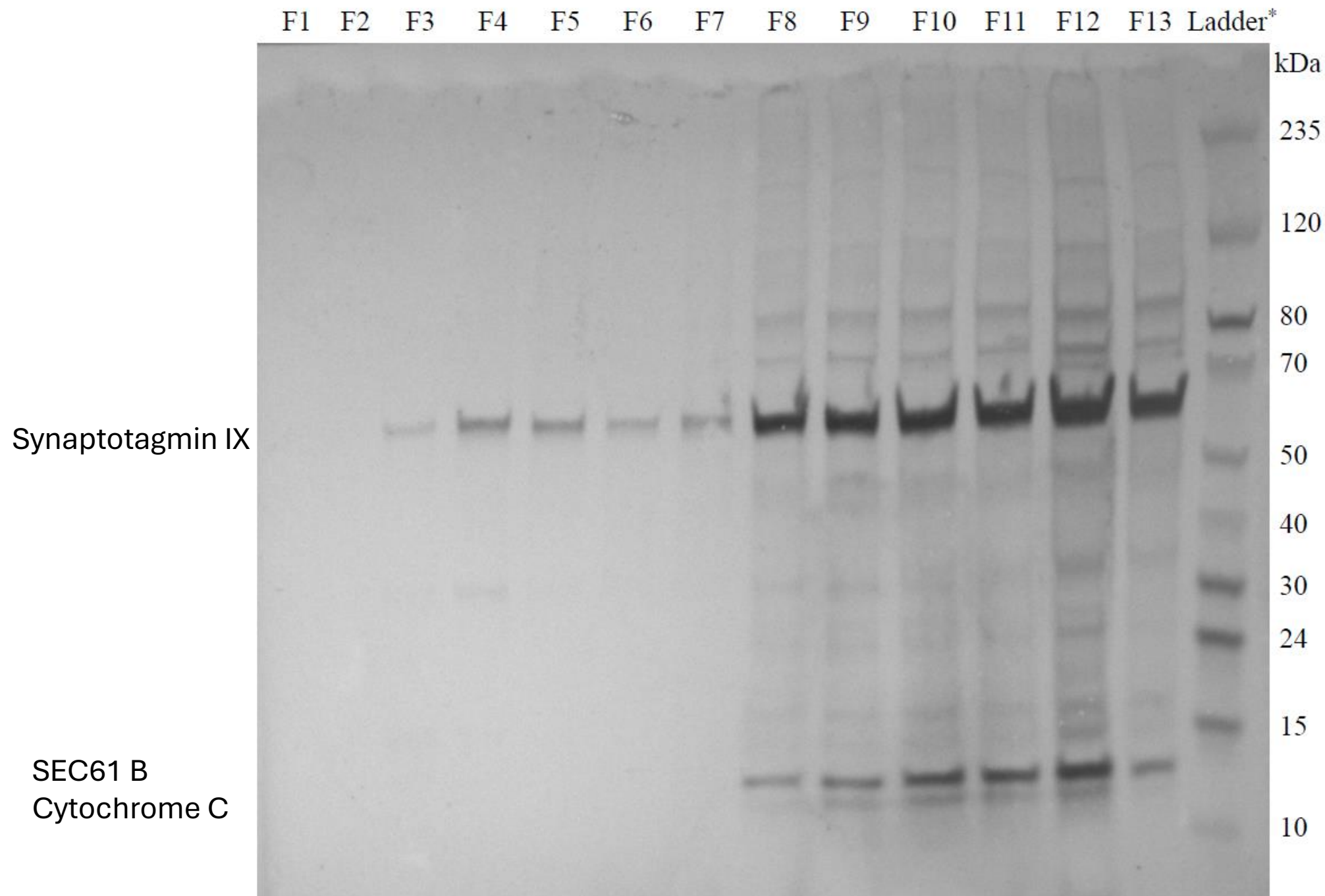

\* Protein ladder (Thermo Fisher Scientific 26634)

Supplement: Figure 1—figure supplement 2—source data 1. [file elife-74989-fig1-figsupp2-data1.pdf]

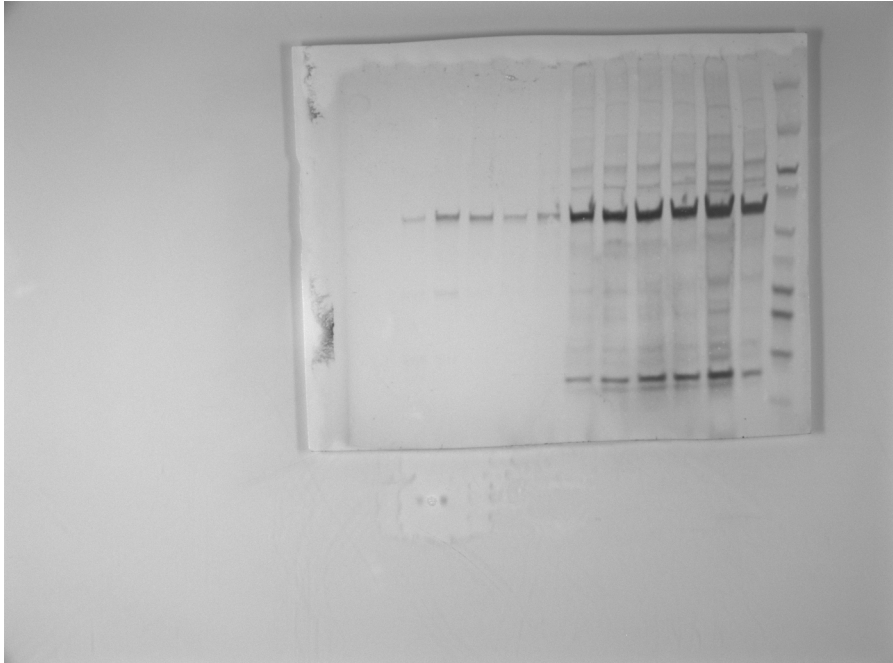

Supplement: Figure 1—figure supplement 2—source data 2. [file elife-74989-fig1-figsupp2-data2.pdf]
